# Supplementary material for: Immobilization of Lipase B from Candida antarctica on Magnetic Nanoparticles Enhances Its Selectivity in Kinetic Resolutions of Chiral Amines with Several Acylating Agents
Source: Life (Basel). 2023 Jul 14;13(7):1560. doi: 10.3390/life13071560 (PMC10381355; doi:10.3390/life13071560)
Supplement: Supplementary file 1 [file life-13-01560-s001.zip › life-2460890-supplementary.pdf]

life

Supplementary Materials

Immobilization of Lipase B from *Candida antarctica* on Magnetic Nanoparticles Enhances the Selectivity of Kinetic Resolutions of Chiral Amines with Several Acylating Agents

Fausto M. W. G. Silva [1], József Szemes [1], Akan Mustashev [1], Orsolya Takács [1], Ali O. Imarah[1,2] and László Poppe [1,3,4,*]

[1] Department of Organic Chemistry and Technology, Budapest University of Technology and Economics, Műegyetem rkp. 3, H-1111 Budapest, Hungary
[2] Chemical Engineering Department, College of Engineering, University of Babylon, 5100 Hilla Babylon, Iraq
[3] Biocatalysis and Biotransformation Research Center, Faculty of Chemistry and Chemical Engineering, Babeş-Bolyai University of Cluj-Napoca, Arany János Str. 11, RO-400028 Cluj-Napoca, Romania
[4] SynBiocat Ltd., Szilasliget u 3, H-1172 Budapest, Hungary
* Correspondence: poppe.laszlo@vbk.bme.hu; Tel.: +36(1)463-3299

Table of Contents

1 Materials and methods 2
1.1 Materials 2
1.2 Analytical methods 2
2 Preparation of biocatalysts 3
3 Analytical data 5

1 Materials and methods

1.1 *Materials*

Iron (III) chloride hexahydrate, sodium acetate trihydrate, tetraethoxysilane (TEOS), 35% ammonia solution, polyethylene glycols (PEG 400, and PEG 4000), hexadecyltrimethoxysilane (HdTMOS), 3-(2-aminoethylamino)propyldimethoxymethylsilane (ApDMOMS), sodium dihydrogen phosphate dihydrate, di-sodium hydrogen phosphate heptahydrate, neopentyl glycol diglycidyl ether (NGDE), sodium phosphate, racemic heptane-2-amine, 1-methoxypropan-2-amine, 1-phenylethan-1-amine, 4-phenylbutan-2-amine, [(±)-**1a-d**, respectively], diisopropyl malonate **2A**, isopropyl cyanoacetate **2B**, sodium 2-chloroacetate, triethylbenzylammonium chloride, and acetic anhydride were purchased from Merck (Darmstadt, Germany) or Alfa Aesar Europe (Karlsuhe, Germany). Ethylene glycol, 2-propanol, ethanol, hexane, were purchased from Merck (Darmstadt, Germany). Patosolv® (a mixture of 10-15% 2-propanol and 85-90% ethanol) was a product of Molar Chemicals (Budapest, Hungary).

1.2 *Analytical methods*

The gas chromatographic (GC) analyses were performed as given in Section 2.2.1 of the main article. The methods and retention times are detailed in Table S1. Representative GC results are shown in Fig. S3-Fig. S15. S19, S23.

Table S1. GC methods and retention times for acetamides [(*S*)- and (*R*)-**1*a-d**] and amides with different acylating agents (**AA**) {diisopropyl malonate **2A**, isopropyl cyanoacetate **2B**, isopropyl 2-ethoxyacetate **2C** [(*S*)- and (*R*)-**3(a-d)(A-C)**]}

**Amin**	**AA**	**Temperature program**	**Retention times [min]***			
			(*S*)-**1*a-d**	(*R*)-**1*a-d**	(*S*)-**3(a-d) (A-C)**	(*R*)-**3(a-d) (A-C)**
(±)-**1a**	**2A**	160-170°C, 0.8°C min^{-1}	2.41	2.45	10.05	10.50
	**2B**	160-168°C, 0.8°C min^{-1}	2.41	2.45	7.40	7.83
	**2C**	160-165°C, 0.8°C min^{-1}	2.41	2.45	3.98	4.08
(±)-**1b**	**2A**	110°C, 10 min; 110-190°C, 5°C min^{-1}	4.84	5.73	20.96	21.67
	**2B**	110°C, 10 min; 110-190°C, 5°C min^{-1}	4.84	5.73	18.14	19.67
	**2C**	110°C, 10 min; 110-140°C, 5°C min^{-1}	4.84	5.73	11.62	13.35
(±)-**1c**	**2A**	160-184°C, 0.8°C min^{-1}	5.85	5.95	21.88	22.94
	**2B**	160-178°C, 0.8°C min^{-1}	5.85	5.95	18.34	19.13
	**2C**	160-169°C, 0.8°C min^{-1}	5.85	5.95	9.07	9.46
(±)-**1d**	**2A**	130°C, 60 min; 130-140°C, 2°C min^{-1}; 140-190°C, 10°C min^{-1}; 190°C, 20 min	65.48	65.97	85.13	85.70
	**2B**	130°C, 60 min; 130-140°C, 2°C min^{-1}; 140-190°C, 10°C min^{-1}; 190°C, 20 min	65.48	65.97	80.62	81.05
	**2C**	130°C, 60 min; 130-140°C, 2°C min^{-1}; 140-190°C, 10°C min^{-1}; 190°C, 5 min	65.48	65.97	71.73	72.07

*The acetamides 1*a-d were prepared by derivatizing the residual amines 1a-d in the samples from the reactions with acetic anhydride.

2 Preparation of biocatalysts

Preparation of the MNP carriers, including the preparation of the magnetite core and its surface modifications, and further immobilization of Lipase B from *Candida antarctica* (CaLB) was based on previously published methods [8].

The surface morphology of the samples was investigated with a JEOL JSM-5500LV scanning electron microscope (SEM). An electron beam energy of 15 kV was used for the investigations. SEM images of MNPs-free form [Fig. S1 (**a**)], and CaLB-MNPs, MNPs-CaLB-bounded form [Fig. S1 (**b**), (**c**), (**d**)] in three different zoom definitions were investigated.

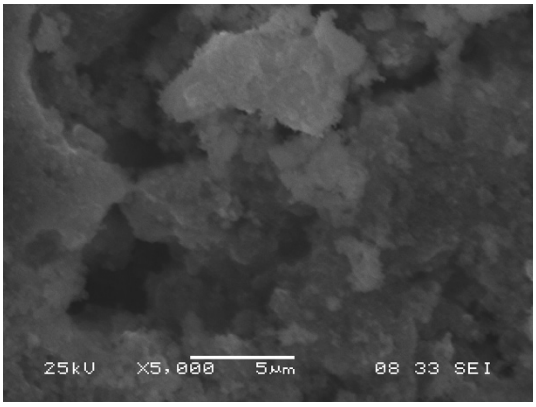

(**a**)

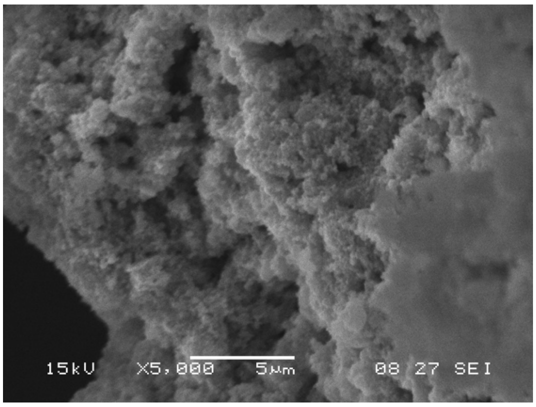

(**b**)

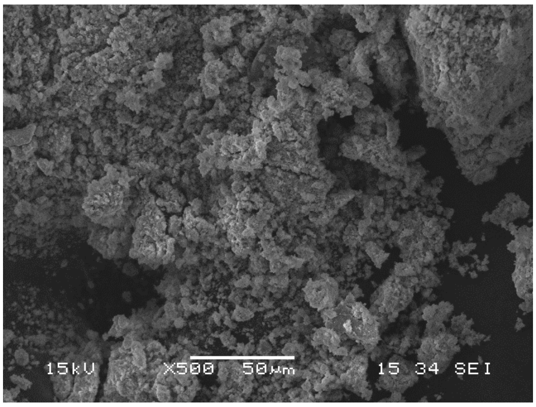

(**c**)

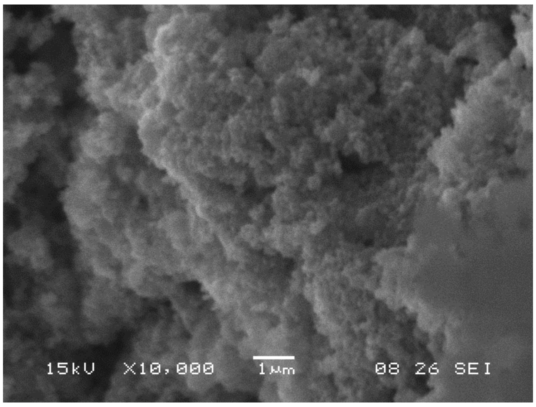

(**d**)

Figure S1. Scanning electron microscope (SEM) analysis of (**a**) MNPs, 5 µm, and (**b**) CaLB-MNPs, 5 µm, (**c**) 50 µm, and (**d**) 1 µm

life

3 Reactor unit

A picture of the thermosttated MNP-reactor with the reactor, syringe pump, and heat exchanger device can be seen in Figure S2.

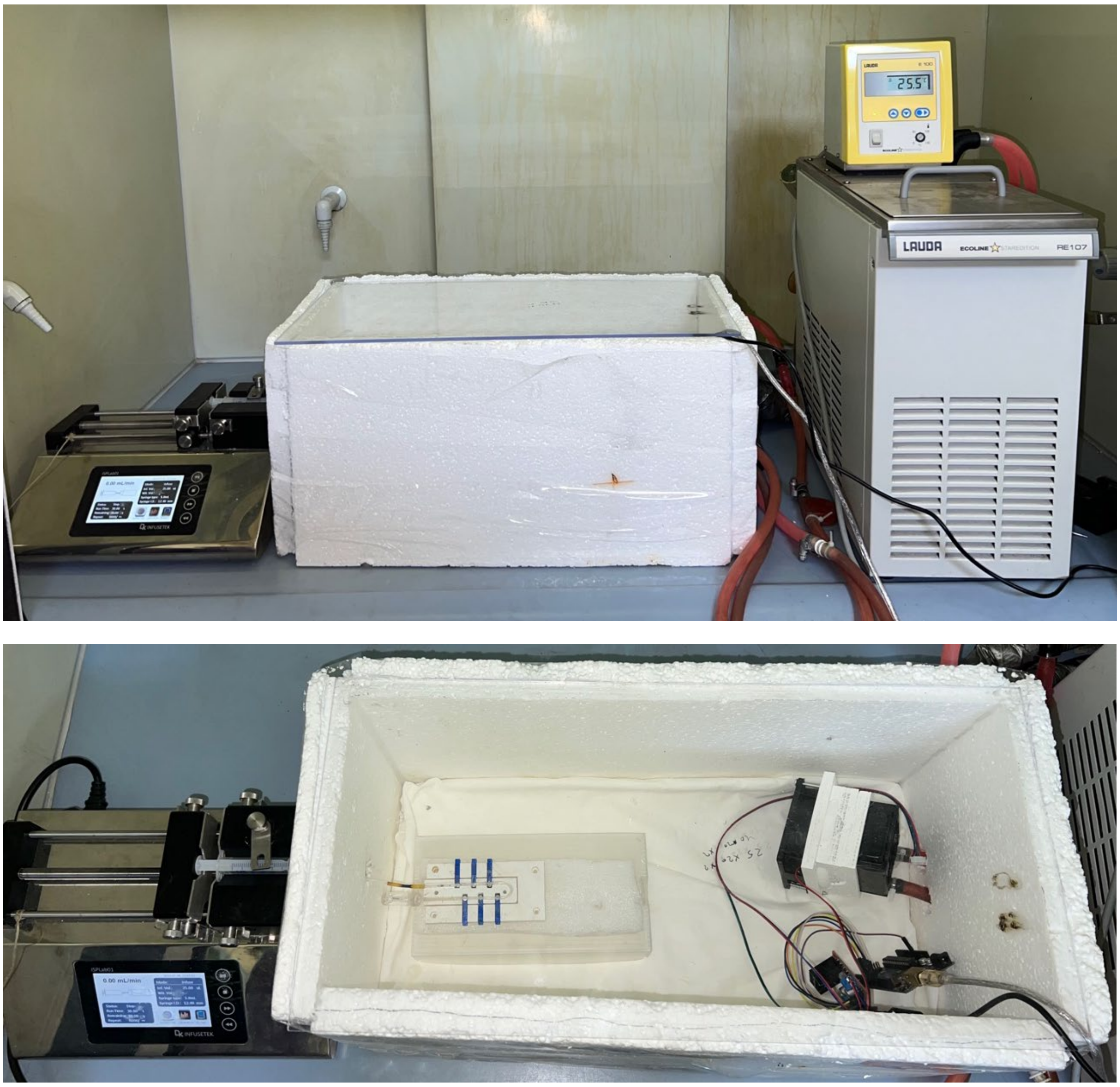

Figure S2. Thermosttated MNP-reactor unit in the frontal and upper view

4 Analytical data

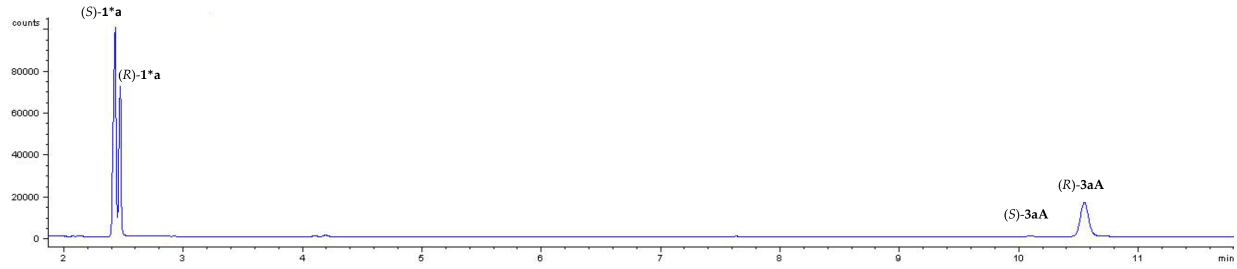

Figure S3. GC chromatogram of the mixture after CaLB-MNPs-catalyzed kinetic resolution of racemic (±)-heptane-2-amine (±)-**1a** with diisopropyl malonate **2A** followed by derivatization with Ac_2O

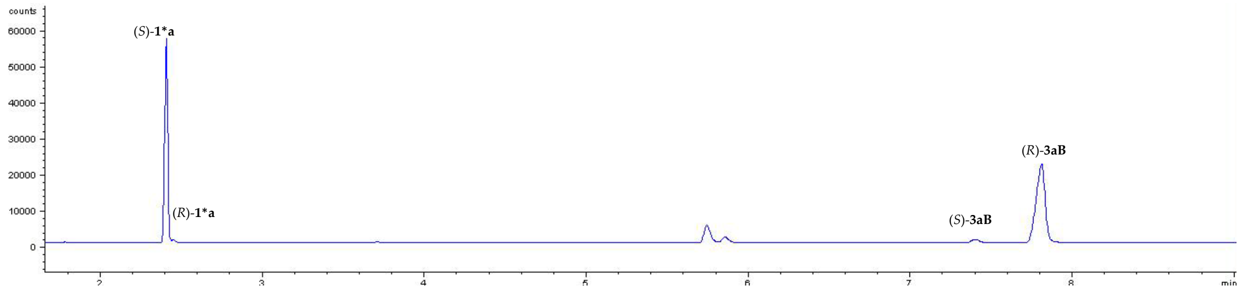

Figure S4. GC chromatogram of the mixture after CaLB-MNPs-catalyzed kinetic resolution of racemic (±)-heptane-2-amine (±)-**1a** with isopropyl 2-cyanoacetate **2B** followed by derivatization with Ac2O

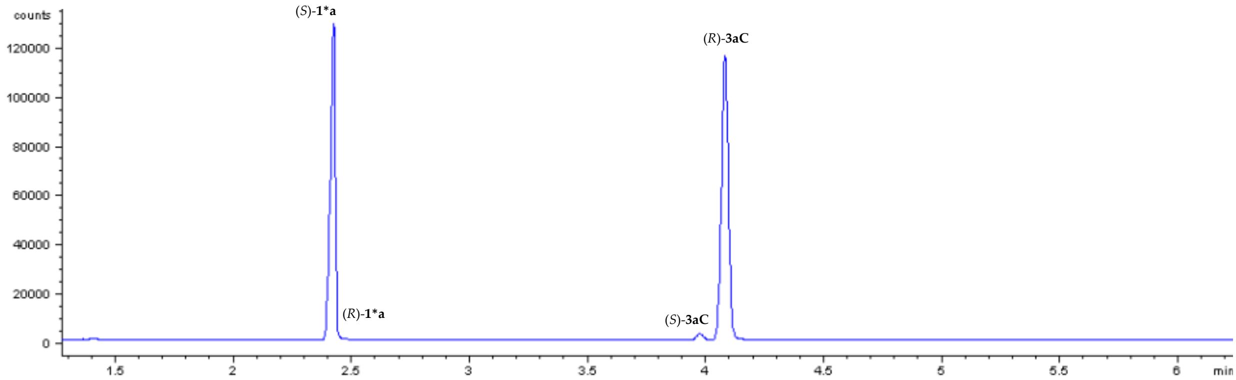

Figure S5. GC chromatogram of the mixture after CaLB-MNPs-catalyzed kinetic resolution of racemic (±)-heptane-2-amine (±)-**1a** with isopropyl 2-ethoxyacetate **2C** followed by derivatization with Ac_2O

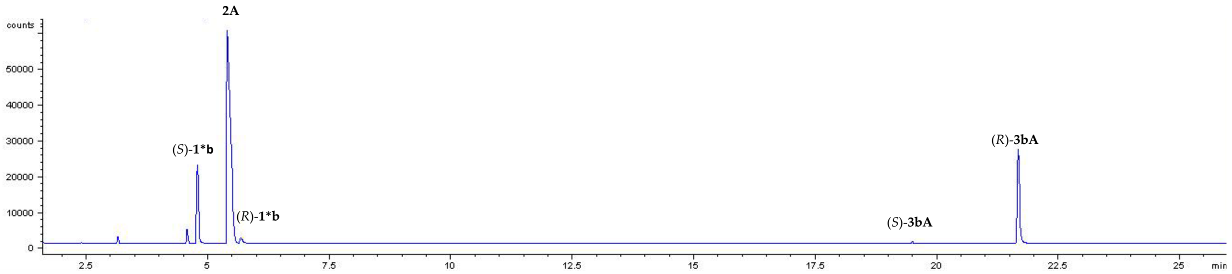

Figure S6. GC chromatogram of the mixture after CaLB-MNPs-catalyzed kinetic resolution of racemic (±)-1-methoxy-2-propan-1-amine (±)-**1b** with diisopropyl malonate **2A** followed by derivatization with Ac_2O

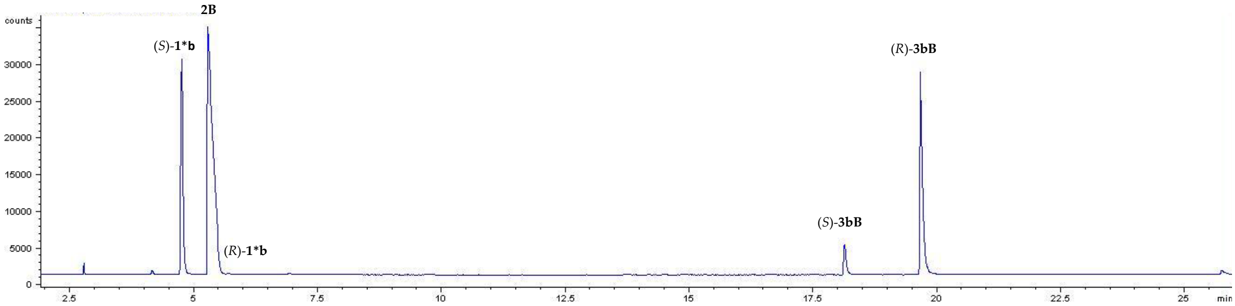

Figure S7. GC chromatogram of the mixture after CaLB-MNPs-catalyzed kinetic resolution of racemic (±)-1-methoxy-2-propan-1-amine (±)-**1b** with isopropyl cyanoacetate **2B** followed by derivatization with Ac_2O

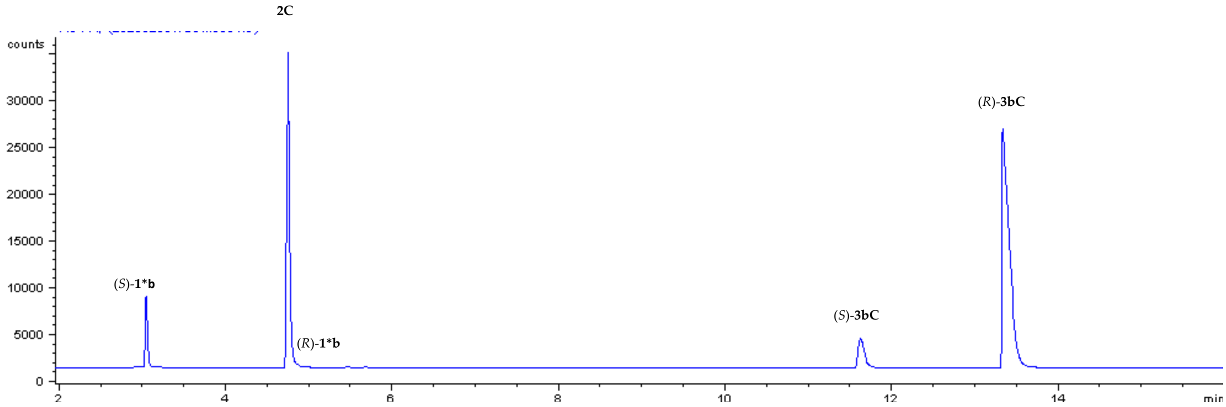

Figure S8. GC chromatogram of the mixture after CaLB-MNPs-catalyzed kinetic resolution of racemic (±)-1-methoxy-2-propan-1-amine (±)-**1b** with isopropyl 2-ethoxyacetate **2C** followed by derivatization with Ac_2O

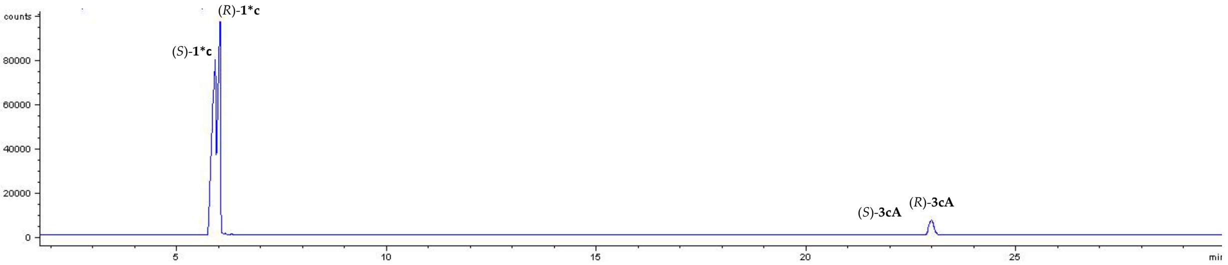

Figure S9. GC chromatogram of the mixture after CaLB-MNPs-catalyzed kinetic resolution of racemic (±)-1-phenylethan-1-amine (±)-**1c** with diisopropyl malonate **2A** followed by derivatization with Ac_2O

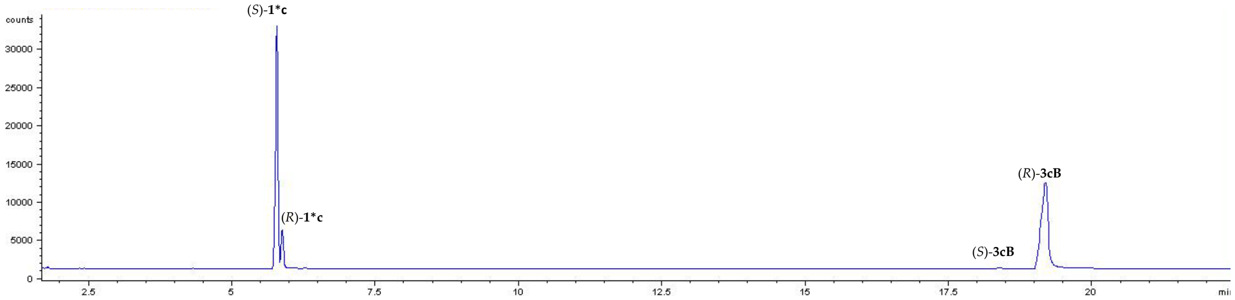

Figure S10. GC chromatogram of the mixture after CaLB-MNPs-catalyzed kinetic resolution of racemic (±)-1-phenylethan-1-amine (±)-**1c** with isopropyl cyanoacetate **2B** followed by derivatization with Ac_2O

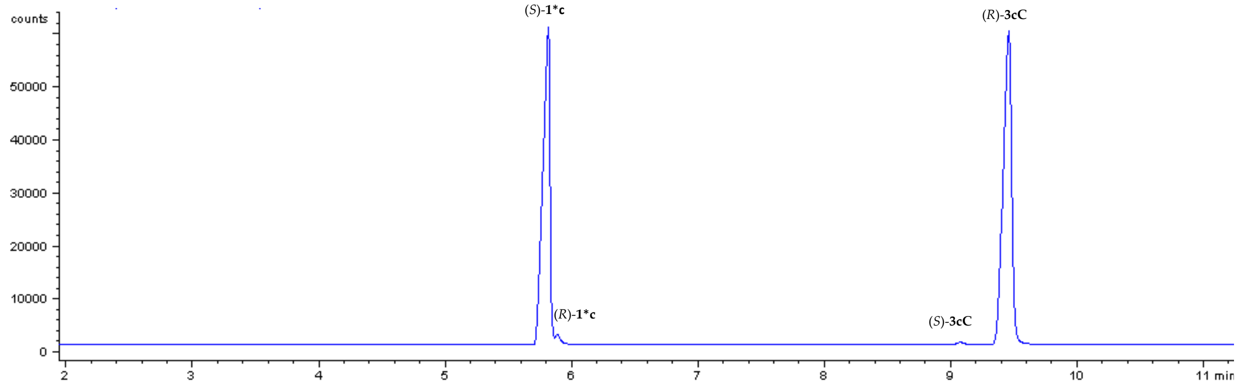

Figure S11. GC chromatogram of the mixture after CaLB-MNPs-catalyzed kinetic resolution of racemic (±)-1-phenylethan-1-amine (±)-**1c** with isopropyl 2-ethoxyacetate **2C** followed by derivatization with Ac_2O

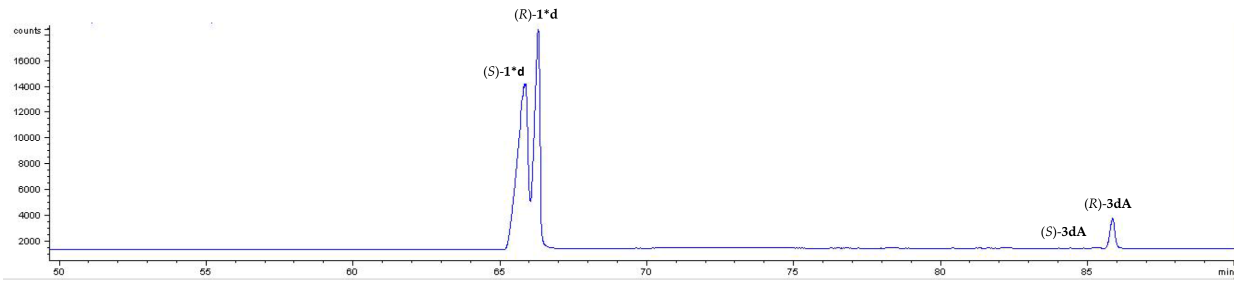

Figure S12. GC chromatogram of the mixture after CaLB-MNPs-catalyzed kinetic resolution of racemic (±)-4-phenylbutan-2-amine (±)-**1d** with diisopropyl malonate **2A** followed by derivatization with Ac_2O

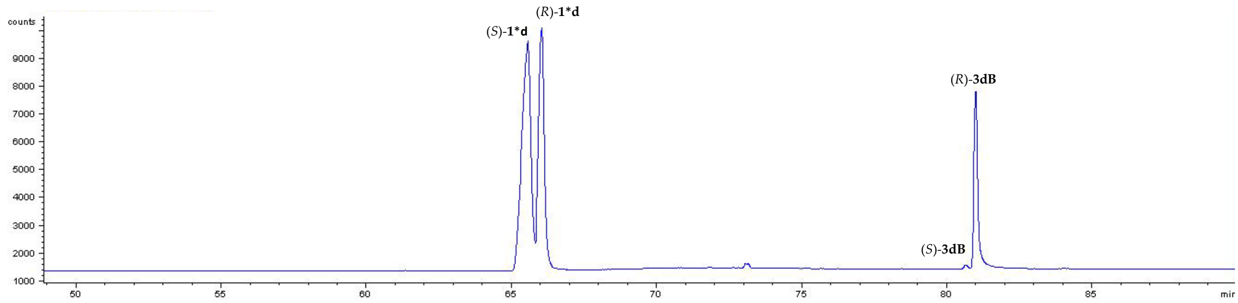

Figure S13. GC chromatogram of the mixture after CaLB-MNPs-catalyzed kinetic resolution of racemic (±)-4-phenylbutan-2-amine (±)-**1d** with isopropyl cyanoacetate **2B** followed by derivatization with Ac_2O

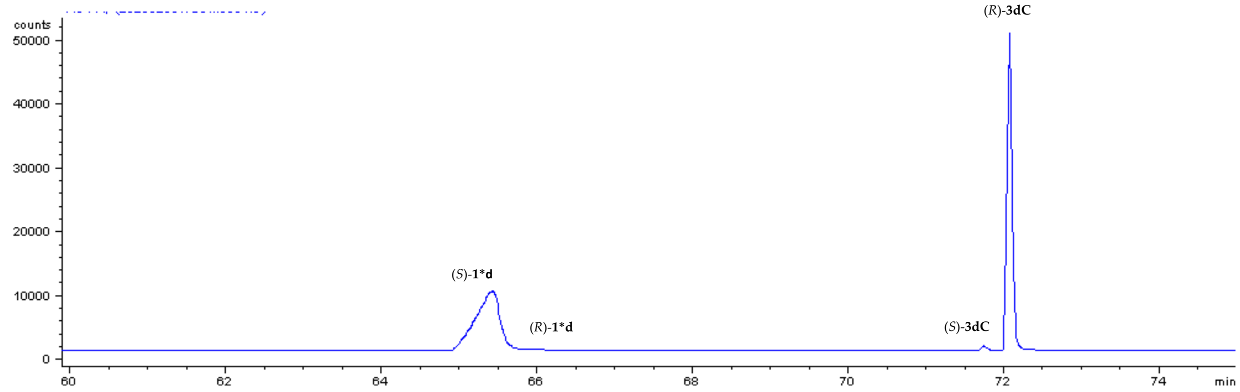

Figure S14. GC chromatogram of the mixture after CaLB-MNPs-catalyzed kinetic resolution of racemic (±)-4-phenylbutan-2-amine (±)-**1d** with isopropyl 2-ethoxyacetate **2C** followed by derivatization with Ac_2O

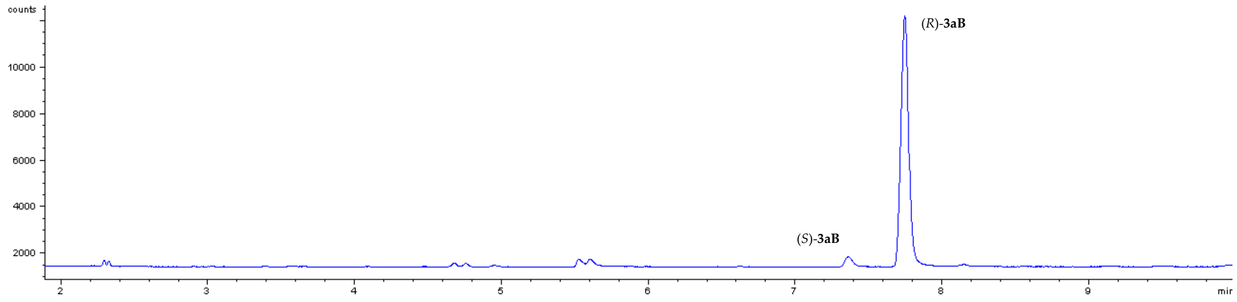

Figure S15. Gas chromatogram of the (*R*)-2-cyano-*N*-(2-heptanyl)acetamide (*R*)-**3aB** produced by CaLB-MNP-catalyzed kinetic resolution in batch mode

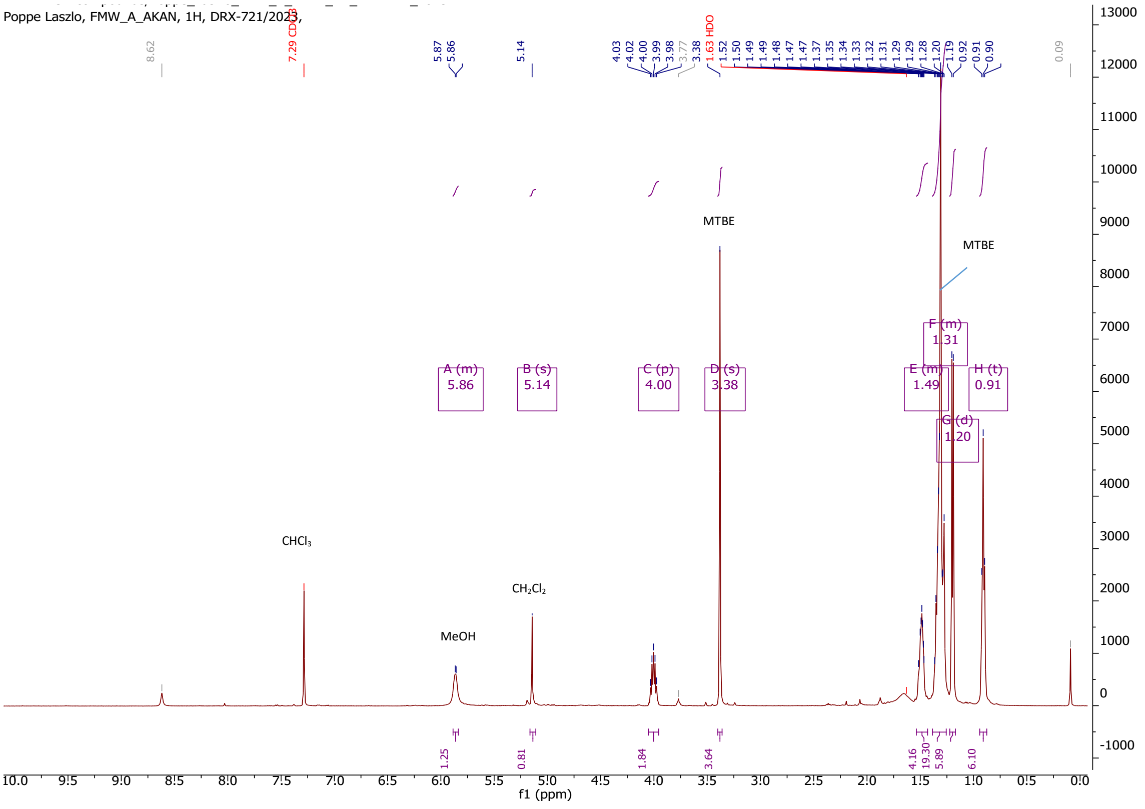

Figure S16. ^{1}H NMR spectrum of the (*R*)-2-cyano-*N*-(2-heptanyl)acetamide (*R*)-**3aB** produced by CaLB-MNP-catalyzed kinetic resolution in batch mode

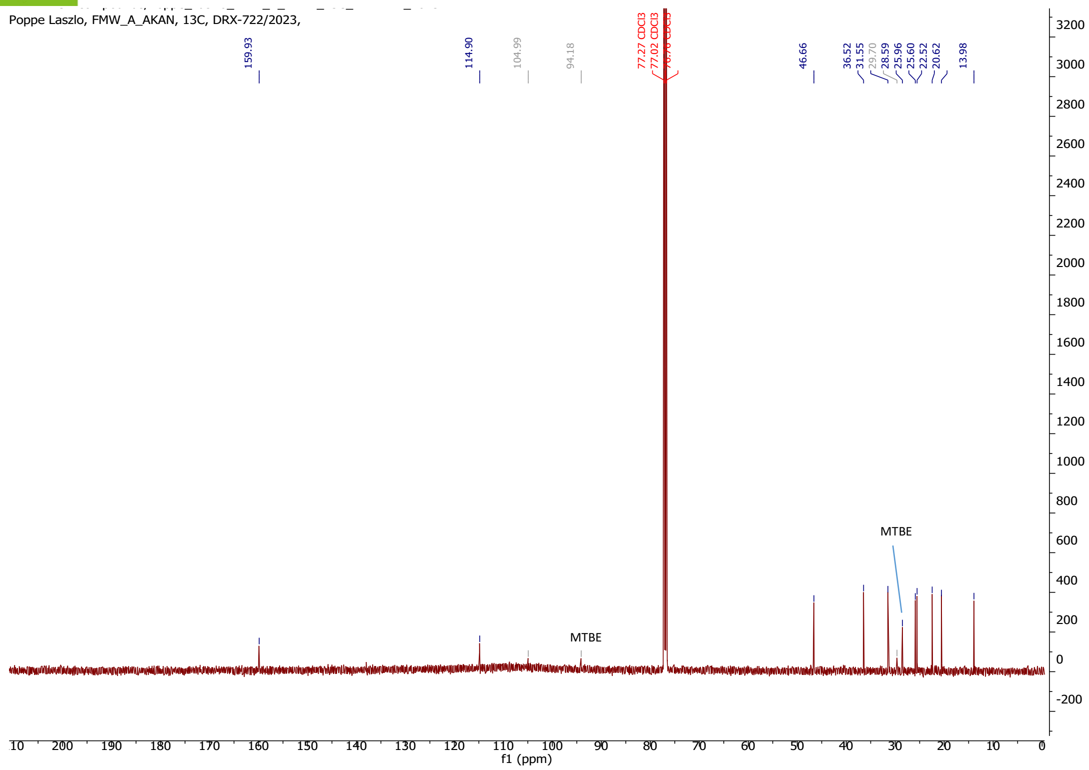

Figure S17. ^{13}C NMR spectrum of the (*R*)-2-cyano-*N*-(2-heptanyl)acetamide (*R*)-**3aB** produced by CaLB-MNP-catalyzed kinetic resolution in batch mode

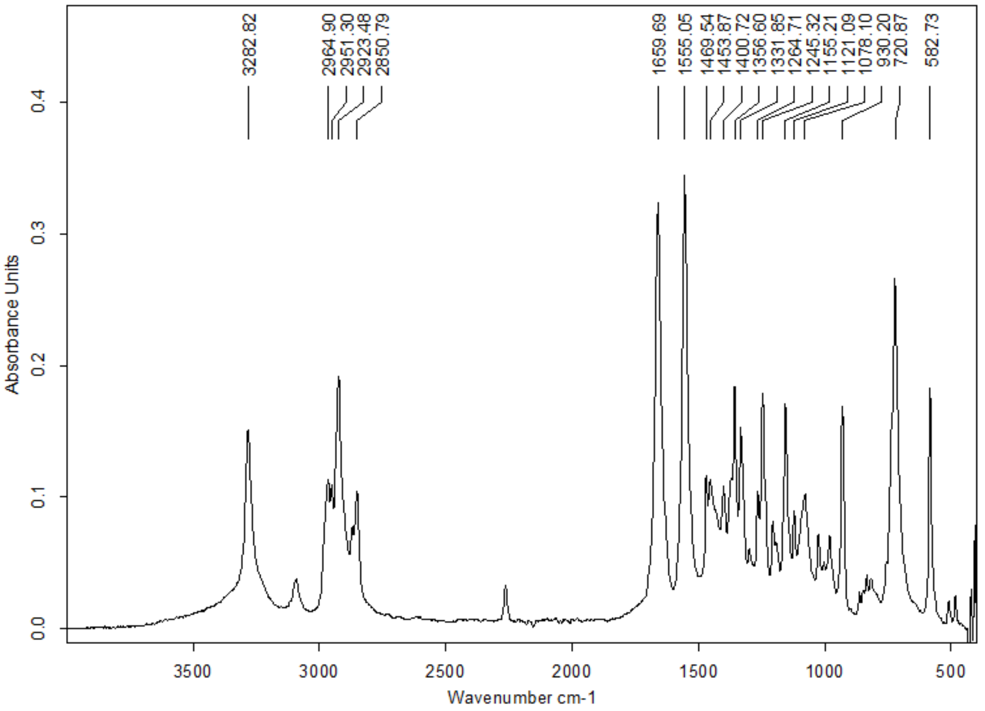

Figure S18. IR spectrum of the (*R*)-2-cyano-*N*-(2-heptanyl)acetamide (*R*)-**3aB** produced by CaLB-MNP-catalyzed kinetic resolution in batch mode.

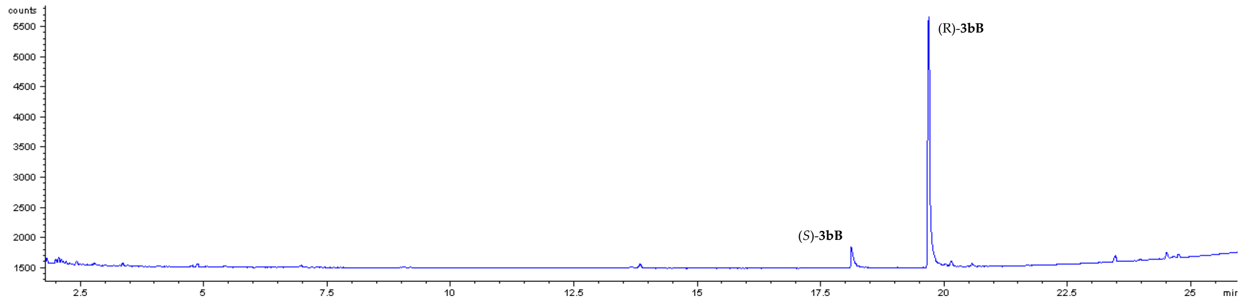

Figure S19. Gas chromatogram of the (*R*)-2-cyano-*N*-(1-methoxy-2-propanyl)acetamide (*R*)-**3bB** produced by CaLB-MNP-catalyzed kinetic resolution in batch mode

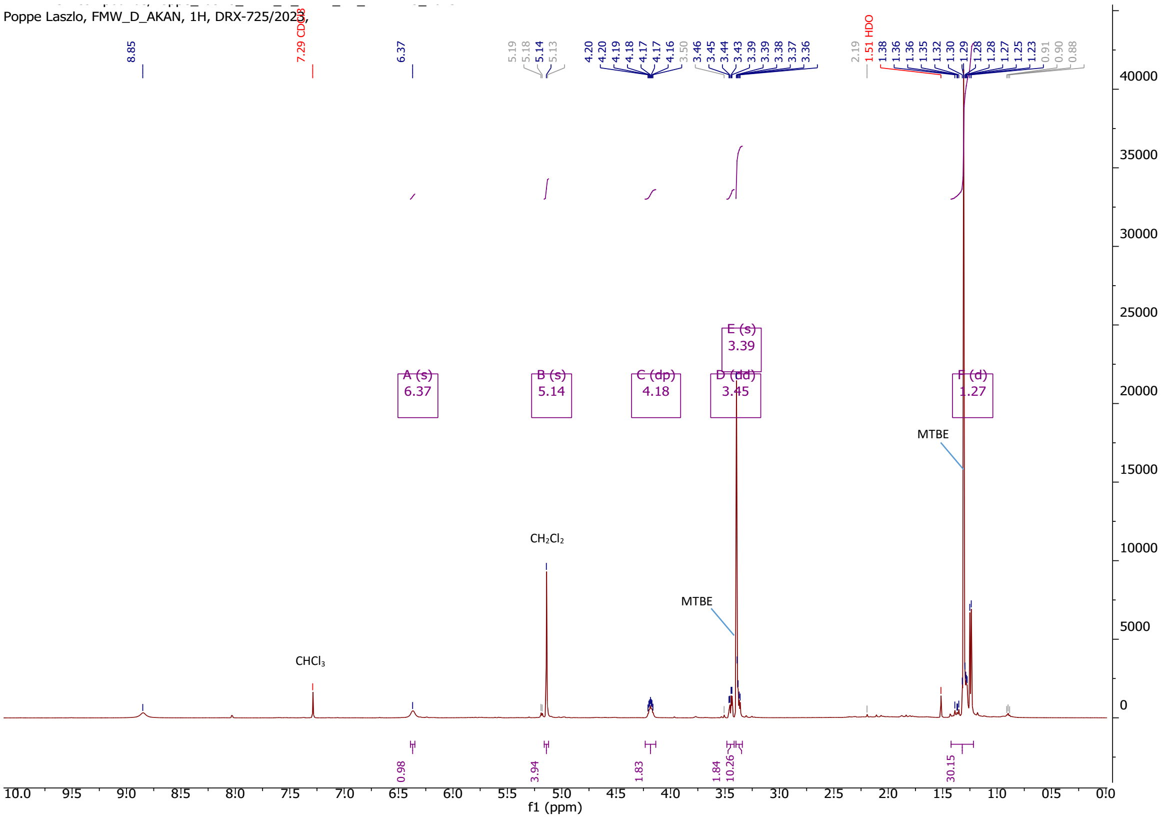

Figure S20. ^{1}H NMR spectrum of the (*R*)-2-cyano-*N*-(1-methoxy-2-propanyl)acetamide (*R*)-**3bB** produced by CaLB-MNP-catalyzed kinetic resolution in batch mode

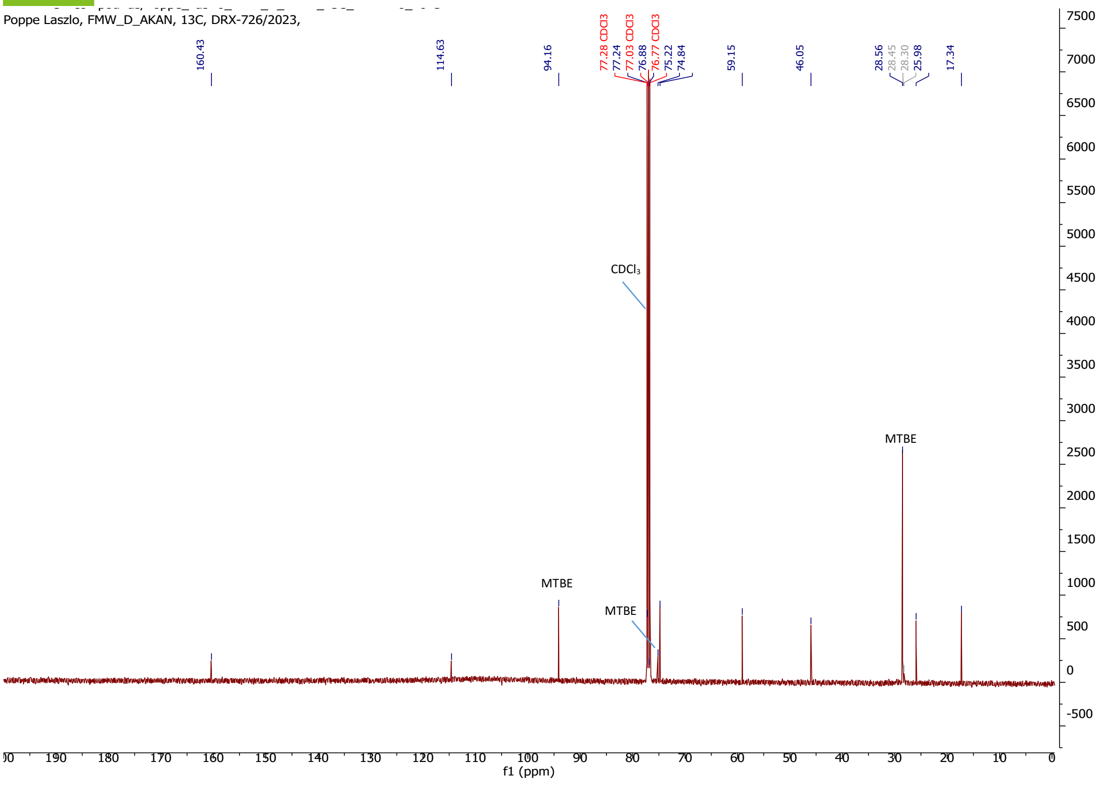

Figure S21. ^{13}C NMR spectrum of the (*R*)-2-cyano-*N*-(1-methoxy-2-propanyl)acetamide (*R*)-**3bB** produced by CaLB-MNP-catalyzed kinetic resolution in batch mode

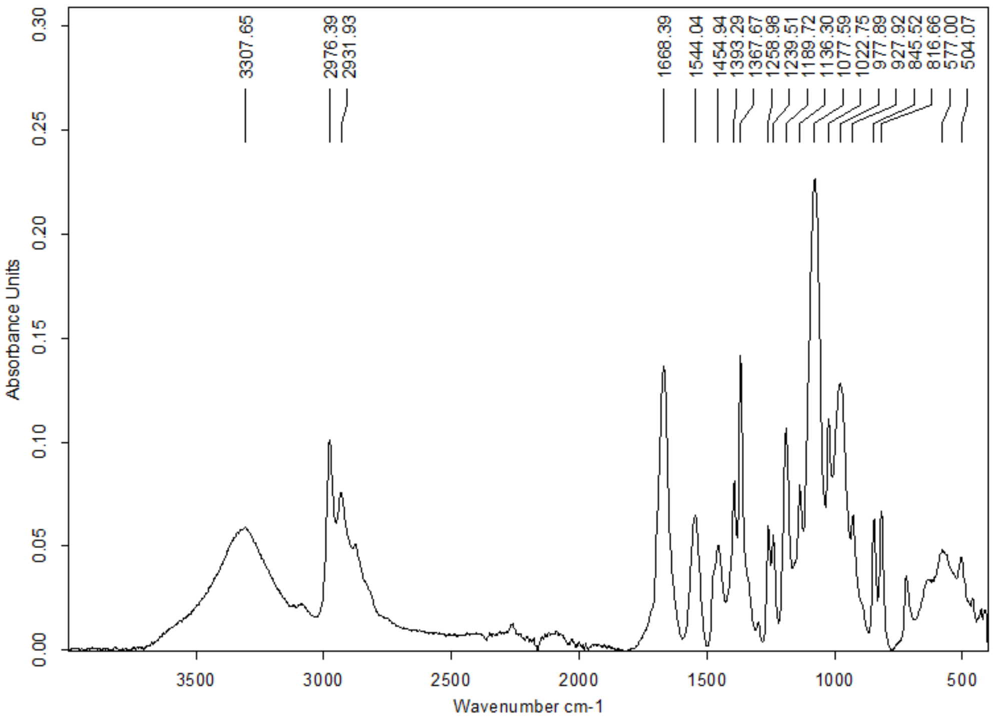

Figure S22. IR spectrum of the (*R*)-2-cyano-*N*-(1-methoxy-2-propanyl)acetamide (*R*)-**3bB** produced by CaLB-MNP-catalyzed kinetic resolution in batch mode.

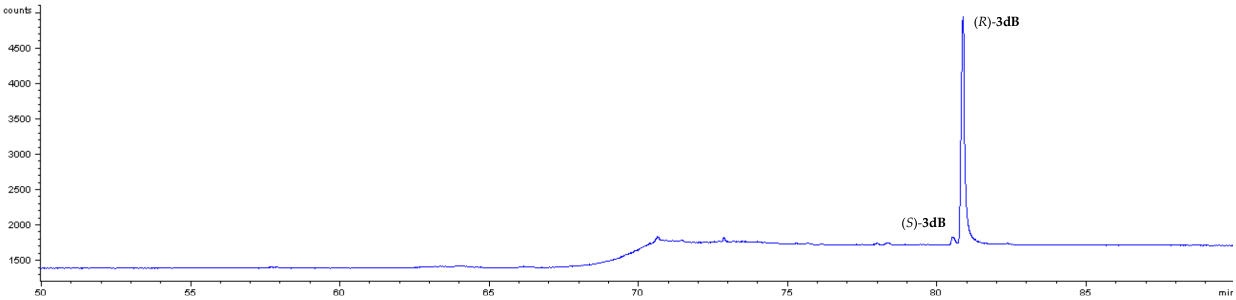

Figure S23. Gas chromatogram of the (*R*)-2-cyano-*N*-(4-phenyl-2-butanyl)acetamide (*R*)-**3dB** produced by CaLB-MNP-catalyzed kinetic resolution in batch mode

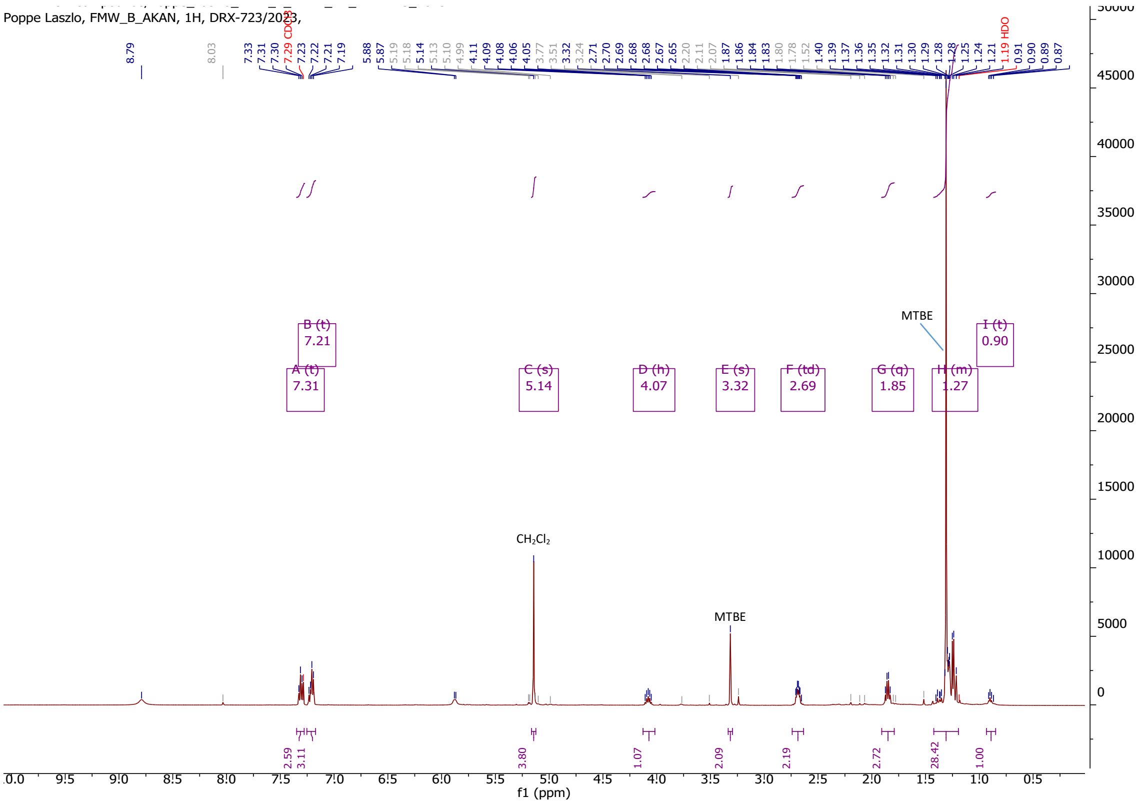

Figure S24. ^{1}H NMR spectrum of the (*R*)-2-cyano-*N*-(4-phenyl-2-butanyl)acetamide (*R*)-**3dB** produced by CaLB-MNP-catalyzed kinetic resolution in batch mode

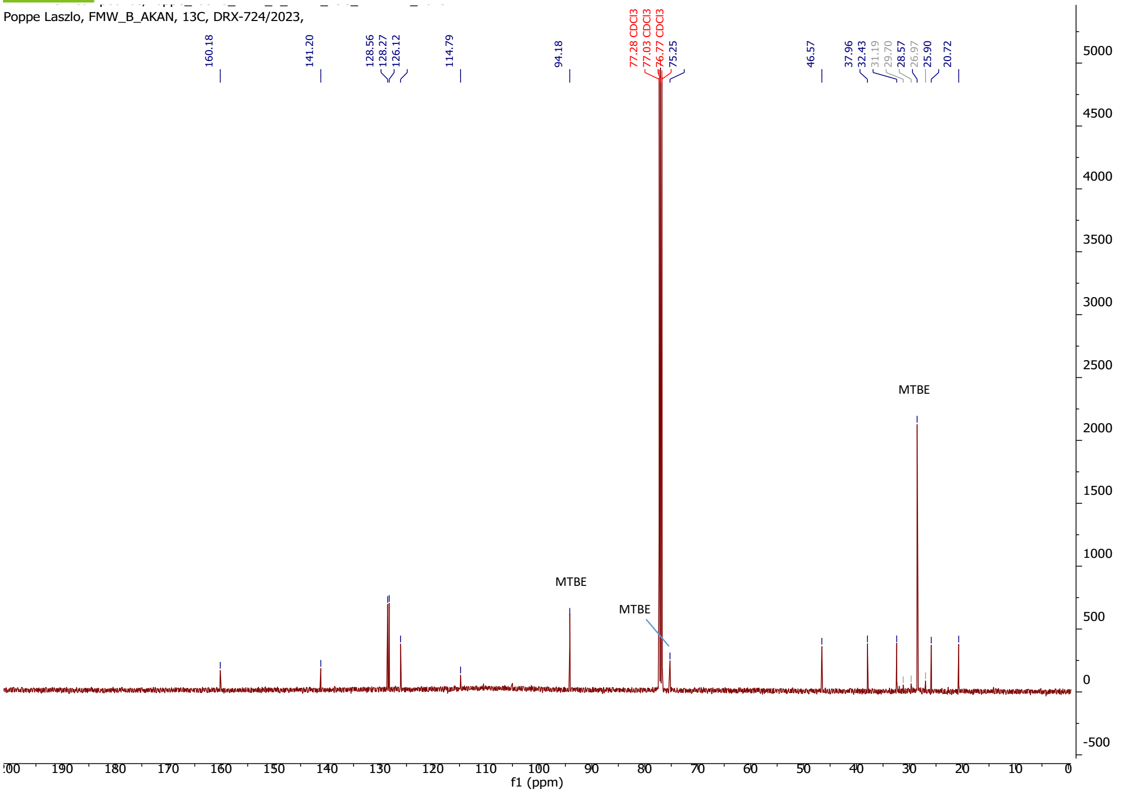

Figure S25. ^{13}C NMR spectrum of the (*R*)-2-cyano-*N*-(4-phenyl-2-butanyl)acetamide (*R*)-**3dB** produced by CaLB-MNP-catalyzed kinetic resolution in batch mode

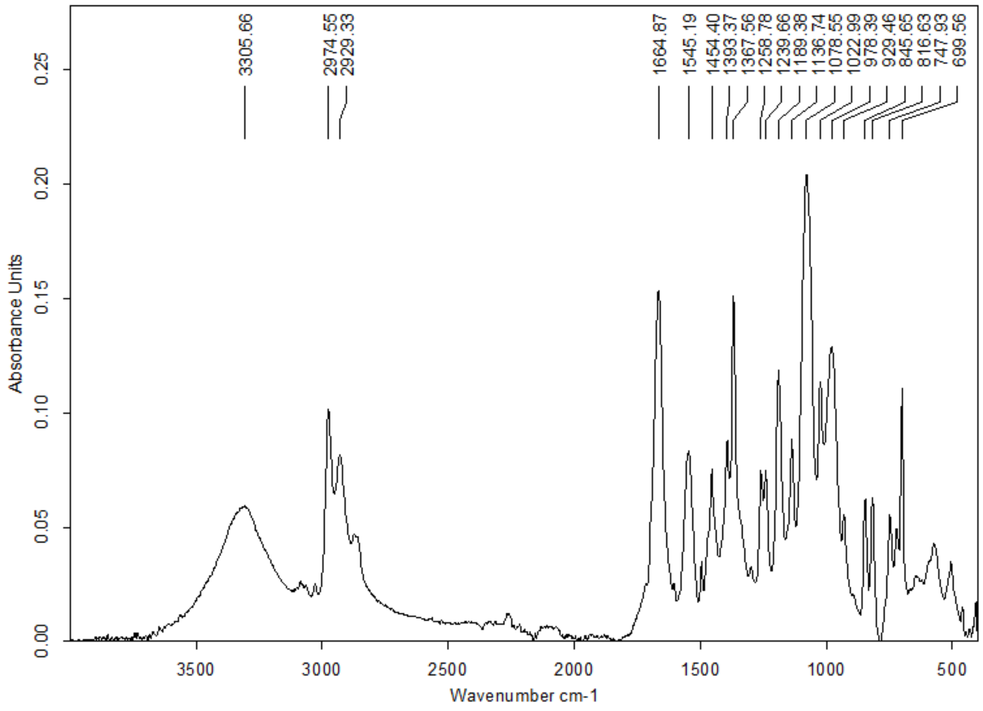

Figure S26. IR spectrum of the (*R*)-2-cyano-*N*-(4-phenyl-2-butanyl)acetamide (*R*)-**3dB** produced by CaLB-MNP-catalyzed kinetic resolution in batch mode.

Supplementary references

8. Imarah, A.O.; Silva, F.M.W.G.; Tuba, L.; Malta-Lakó, Á.; Szemes, J.; Sánta-Bell, E.; Poppe, L. A Convenient U-Shape Microreactor for Continuous Flow Biocatalysis with Enzyme-Coated Magnetic Nanoparticles-Lipase-Catalyzed Enantiomer Selective Acylation of 4-(Morpholin-4-yl)butan-2-ol. *Catalysts* **2022**, *12*, 1065. doi:10.3390/catal12091065
